# Treatment evolution in spinal muscular atrophy: insights from the SMArtCARE registry

**DOI:** 10.1093/brain/awaf472

**Published:** 2025-12-23

**Authors:** Cornelia Voigt-Müller, Michelle Pfaffenlehner, Günther Bernert, Hakan Cetin, Tim Hagenacker, Heike Kölbel, Hanns Lochmüller, Christian Pfeuffer, Katharina Vill, Maggie C Walter, Janbernd Kirschner, Astrid Pechmann, SMArtCARE study group, Pascal Martin, Christoph Neuwirth, Andreas Merkenschlager, René Günther, Mathias Müller, Regina Trollmann, Gert Wiegand, Sabine Illsinger, Omar Keritam, Matthias Baumann, Sarah Bernsen, Claudia Weiß, Gerd Meyer zu Hörste, Matthias Türk, Daniel Zeller, Thomas M K Völkl, Uta Diebold, Verena Haug, Wolfgang Löscher, Dorothea Holzwarth, Mareike Schimmel, Burkhard Stüve, Karin Wabnegger, Ralf A Husain, Oliver Schwartz, Matthias Vorgerd, Eva Matzker, Gilbert Wunderlich, Raffi Topakian, Eckard Hamelmann, Anette Schwerin-Nagel, Marina Flotats-Bastardas, Astrid Blaschek, Christof Reihle, Astrid Bertsche, Ruth Janßen, Andreas Merkenschlager, Sandra Nebgen, Martin Smitka, Andreas Ziegler, Manuela Theophil, Ruth Janßen, Moritz Metelmann, Birgit Kauffmann, Andreas Hahn, Gudrun Schreiber, Anette Schwerin-Nagel, Astrid Eisenkölbl, Martin Winterholler, Eva Stögmann, Veronka Horber, Friedrich Ebinger, Tobias Geis, Martin Fleger, Jessika Johannsen, Meike Bettina Göricke, Veronika Pilshofer, Maren Fuchs, Paul Lingor, Alexander Mensch, Robert Steinbach, Sebastian Friedrich, Julian Großkreutz, Zeljko Uzelac, Friederike Sophie Kohlmann, Harald Faninger, Simone Mahal, Christoph Korenke, Harald Binder, Katharina Dörnbrack, Jasmin Bischofberger, Janina Gburek-Augustat, Hanna Sophie Lapp, Tanja Zindler, Bettina Behring, Miriam Hiebeler, Hans Hartmann, Benjamin Stolte, Bernhard Fasching, Christian Lechner, Patrick Weydt, Joanna Schneider, Sarah Wiethoff, Julia Bellut, Johannes C Stoffels, Antje Schmidt, Corrine G C Horlings, Alexandra Klotz, Daniela Angelova-Toshkina, Sabine Knafl-Slamanig, Daniela Steuernagel, Hélène Guillemot, Melanie Drabant, Kathrin Hornung, Hormos Salimi Dafsari, Petra Müller, Georg Classen, Michael Grässl, Viola Horneff, Sarah Braun, Josefine Lendeckel, Oliver Summ, Janina Gburek-Augustat, Ilona Krauspe-Stübecke, Maja von der Hagen, Michal Fischer, Klaus Goldhahn, Oliver Summ, Christa-Caroline Bergner, Adela Della Marina, Marieke Ziegler, Omar Keritam, Kyriakos Martakis, Bernd Wilken, Michael Grässl, Manuel Pühringer, Christophe Rauch, Hanna Küpper, Julia Maren Lüttgenau, Marion Pilz, Jonas Denecke, Melanie Haage-Brüning, Kathrin Mörtlbauer, Franziska Grünberg-Lemi, Marcus Deschauer, Caroline Deborah Stapf, Annekathrin Rödiger, Cornelia Voigt-Müller, Janina von der Gablentz, Beate Muschner, Ronny Nebelung, Alina Sapranidis, Magdalena Gosk - Tomek, Heike Losch, Maren Hackenberg, Alexandra Giese, Ursula Schneider Rosinger, Sybille Döhler, Maria Rehfeldt, Stephanie Schüssler, Stephan Wenninger, Barbara Ramadan, Svenja Neuhoff, Gudrun Zulehner, Rachel Fabian, Angela Kaindl, Johannes Flammer, Brigitte Brauner, Carmen Hollerauer, Tajana Brunckhorst, Anna Hotter, Gabriel Dworschak, Lisa Jung, Sabine Borowski, Timo Deba, Anne-Katrin Güttsches, Lisa Starke, Jürgen-Christoph von Kleist-Retzow, Ina Krahwinkler, Burkhard Gess, Lukas Neubauer, Michael Zemlin, Wolfgang Müller-Felber, Markus Blankenburg, Josefin Grabow, Philipp Hoth, Ilka Lehnert, Georg Friedrich Hoffmann, Arpad von Moers, Philipp Hoth, Petra Baum, Nina Rademacher, Ulf Hustedt, Bernhard Fasching, Jolanta Heinzinger, Magnus Ruf, Barbara Plecko, Marie-Luise Drax, Frank Kerling, Nadja Kaiser, Eleni Chatzizisi, Joenna Driemeyer, Meret Tabea Dreyer, Elke Pernegger, Isabell Cordts, Ilka Schneider, Benjamin Ilse, Sara-Maria Simon, Bernd Friedrich, Kurt Wollinsky, Janina Gburek-Augustat, Franz-Philipp Brändle, Anna Wiesenhofer, Iris Marquardt, Clemens Schächter, Christine Mauz, Christina Knellwolf, Maren Freigang, Raphael Seebröker, Simone Thiele, Melina Schlag, Jakob Rath, Margrit Koglin, Lisa Valerie Bitzan, Christine Leypold, Florian Junge, Katrin Schüler, Franziska Busch, Sabrina Geissler, Heike de Vries, Katja Köbbing, Ute Weyen, Ioanna Angeli, Barbara Plecko, Kerstin Böcking, Moritz Tacke, Michael Schroth, Frauke Ehrhardt, Monika Meyer, Nicole Claus, Stefan Kölker, Kathrin Bühner, Monika Meyer, Jennifer Rolack, Britta Holtkamp, Inka Brandes, Jakob Rath, Rahel Schuler, Elisabeth Steiner, Martin Drechsel, Dorothee Fütterer, Patricia Müller, Paula Steffens, Ulrike Wolf, Tanja Neumair, Marianne Rösner, Anna Katharina Koelsch, Almut Fritsch, Eva Jung, Clemens Runge, Kornelia Kreiser, Andreas Merkenschlager, Susanne Grinzinger, Yvonne Lechner, Renate Peters, Max Behrens, Adrian Tassoni, Zylfije Dibrani, Marian Kollaske, Laura Stimmer, Jaquelina Lipka, Martin Krenn, Martina Delitz, Alexandra Wagner, Katja D’Amico-Hofmann, Anna Resch, Lisa Allenstein, Stephanie Molitor, Peter Huppke, Andor Horváth, Jakob Wefers, Julia Turowski, Sabine Hettrich, Iris Hannibal, Lutz Dondit, Aglaia Lütjens, Stefan Kappel, Sandy Förster, Sabine Specht, Katharin Müller-Kaempffer, Stefan Kappel, Barbara Andres, Krisitin Kook, Martin Krenn, Andrea Hackemer, Doris Roland-Schäfer, Ingo Conze, Deike Weiss, Mario Müller, Anna Elmecker, Petra Rau, Thomas Kendzierski, Uta Smesny, Nikolai Jung, Astri Fromm, Johannes Dorst, Henriette Kiep, Christian Rauscher, Katia Vettori, Tobias Linden, Tim Kampowski, Anja Boegner, Eva Malm, Fritz Zimprich, Barbara Schwegmann, Vladimir Dukic, Rike Remmert, Christa Bretschneider, Barbara Fiedler, Corinna Rademacher, Loreen Plugge, Sybille Stephan-Lutter, Lena Manssen, Eva Wendel, Martina Neininger, Anja Meenken, Moritz Niesert, Annika Meenken, Nina Schlaghecke, Meike Kahrs, Fritz Zimprich, Lena Ruß, Eva Jansen, Heidi Starke, Jörg Tiedemann, Verena Angermair, Vincent Gmeiner, Sebastian Plutz, Anna-Lena Fleischer, Sabine Wider, Kristin Loyal, Victoria Göbel, Alexander Bobe, Anna-Maria Gaese, Katharina Längle, Birgitt Moed, Franziska Wenzel, Katharina Wagner, Peter Reilich, Thomas Dörnen, Corinna Stoltenburg, Hanna Reinke-Hermer, Sindy Becker, Eva Johann to Settel, Karsten Krause, Christine Sprengart, Birgit Warken-Madelung, Daniela Banholzer, Katja Koch, Urania Kotzaeridou, Katja Koch, Jana Barluschke, Kirsten Fündeling-Tielitz, Gudrun Zulehner, Katharina Unverfehrt, Esther Maihöfer, Ricarda von Heynitz, Max Obenauf, Nadin Fedtke, Juliane Hug, Mohammad Kalaf, Julia Seifert, Ramona Menzel, Mirjam Spitzinger, Anna Hüpper, Shalin Nerkamp, Lukas Nalbach, Beate Schlotter-Weigel, Zoé Messerli, Anja Müller, Geerthe Margriet Balk, Irene Lange, Nicole Groß, Annika Roser-Unruh, Elke Preisendanz, Jessica Ribke, Annegret Hoevel, Jessica Ribke, Corinna Doege, Daniel Bormann, Heidrun Krämer-Best, Birgit Meßmer, Barbara Vogel, David Strube, Saskia Beyer, Matthias Eckenweiler, Maj-Britt Bartels, Aneliya Simeonova Eroglu, Elena Gantschew, Elke Tiefenthaler, Nora Maier, Janina Fürup, David Leibensperger, Laura Kahle, Sibylle Vogt, Sabine Stein

**Affiliations:** Department of Neuropediatrics and Muscle Disorders, Medical Center—University of Freiburg, Faculty of Medicine, University of Freiburg, Freiburg D-79106, Germany; Freiburg Center for Data Analysis and Modeling and AI, University of Freiburg, Freiburg D-70104, Germany; Faculty of Medicine and Medical Center—University of Freiburg, Institute of Medical Biometry and Statistics, Freiburg D-79104, Germany; Neuromuscular Research Division, Department of Paediatrics and Adolescent Medicine, University Hospital Vienna, Vienna A-1090, Austria; Department of Neurology, Medical University of Vienna, Vienna A-1090, Austria; Comprehensive Center for Clinical Neurosciences and Mental Health, Medical University of Vienna, Vienna A-1090, Austria; Department of Neurology, and Center for Translational Neuro- and Behavioral Sciences (C-TNBS), University Medicine Essen, Essen D-45147, Germany; Department of Neuropediatrics and Neuromuscular Centre for Children and Adolescents, University of Duisburg-Essen, Essen D-45147, Germany; Children’s Hospital of Eastern Ontario Research Institute; Division of Neurology, Department of Medicine, The Ottawa Hospital; and Brain and Mind Research Institute, University of Ottawa, Ottawa, ON K1N 6N5, Canada; Deutsche Gesellschaft für Muskelkranke, Freiburg D-79112, Germany; Department of Pediatric Neurology and Developmental Medicine and LMU Center for Children with Medical Complexity, Dr. von Hauner Children’s Hospital, LMU Hospital, Ludwig-Maximilians-University, Munich D-80337, Germany; Friedrich-Baur-Institute, Department of Neurology, Ludwig-Maximilians-University of Munich, Munich D-80336, Germany; Department of Neuropediatrics and Muscle Disorders, Medical Center—University of Freiburg, Faculty of Medicine, University of Freiburg, Freiburg D-79106, Germany; Department of Neuropediatrics and Muscle Disorders, Medical Center—University of Freiburg, Faculty of Medicine, University of Freiburg, Freiburg D-79106, Germany

**Keywords:** spinal muscular atrophy, SMA, DMT, disease-modifying treatment

## Abstract

Real-world treatments for 5q-spinal muscular atrophy (SMA) have evolved rapidly following the sequential approval of three disease-modifying treatments (DMTs): nusinersen, onasemnogene abeparvovec and risdiplam. The aim of this study was to map the sequence and timing of SMA treatments accurately using the SMArtCARE registry, a disease-specific registry for patients with SMA across 84 participating centres in Germany, Austria and Switzerland.

All patients registered in SMArtCARE were included in the analysis. Patients were grouped based on their treatment regimen: those who remained on the first DMT versus those who switched DMT. The impacts of clinical and genetic factors on treatment decisions were evaluated, including age at initiation of treatment, *SMN2* copy number, motor function status, the need for ventilator support or tube feeding, and the presence of scoliosis.

A total of 2140 patients were included. Of these, 1294 patients (60.5%) initiated treatment with nusinersen, 514 patients (24.0%) with risdiplam and 243 patients (11.4%) with onasemnogene abeparvovec. Overall, 1366 patients (63.8%) remained on the first DMT. Most treatment switches occurred shortly after approval of a new DMT. Notably, most patients who switched showed no change in motor milestone status between the start of the first and the second DMT.

In this large real-world cohort, we present the first comprehensive analysis of SMA treatment patterns across all age groups and disease severities. Although most patients remained on the first DMT, switches were observed, mainly after DMT approvals. Decisions to switch appear multifactorial and are not related directly to motor function effectiveness.

**See Okamoto (https://doi.org/10.1093/brain/awag041) for a scientific commentary on this article.**

## Introduction

Spinal muscular atrophy (SMA) is a rare neuromuscular disease caused by bi-allelic pathogenic variants in the *SMN1* gene, leading to progressive proximal muscle weakness.^1^ The clinical spectrum of the disease is broad, ranging from infants with profound muscle weakness who present within the first weeks of life with symptoms such as respiratory insufficiency and failure to thrive because of swallowing difficulties, to ambulatory adults with only a mild proximal muscle weakness and a relatively stable disease course.^2^ Adjacent to *SMN1* lies the *SMN2* gene, which differs from *SMN1* by only a few nucleotides. However, this difference leads to the predominant production of a truncated, non-functional protein, with only a small proportion of functional full-length SMN protein. As a result, *SMN2* acts as a disease modifier, whereby the number of *SMN2* copies is inversely correlated with disease severity.^3^

In recent years, three disease-modifying treatments (DMTs) have been developed and approved by the US Food and Drug Administration (FDA), the European Medicines Agency (EMA) and other regulatory agencies: nusinersen, an antisense oligonucleotide; onasemnogene abeparvovec (OA), a gene addition therapy; and risdiplam, a small molecule. There is robust evidence from clinical trials and real-world data that these DMTs can stabilize the disease course of SMA and even enable improvements in motor function and development not commonly expected without treatment.^4-10^ The treatment response is highest when treatment is initiated in presymptomatic patients.^11-13^ Consequently, newborn screening programmes (NBS) have been implemented in many countries,^14^ including Germany and Austria, since 2021.^15^

The treatment landscape for SMA has continued to change over the past few years, with the sequential approvals of nusinersen, OA and risdiplam (Table 1).

**Table 1:** Overview of the three European Medicines Agency-approved disease-modifying treatments

Nusinersen (antisense oligonucleotide)	Onasemnogene abeparvovec (gene replacement therapy)	Risdiplam (small molecule)
Mechanism		
* SMN2*: mRNA splicing modifier	*SMN1*: addition of functional *SMN1* gene via non-integrating AAV9 vector	*SMN2*: mRNA splicing modifier
Drug approval (European Medicines Agency)		
All patients with 5q-SMA (June 2017)	5q-SMA type 1, or 5q-SMA with up to three *SMN2* copies (May 2020), no formal restriction on age or body weight, but clinical use is limited to infants and young children	5q-SMA type 1–3, or 5q-SMA with up to four *SMN2* copies, aged >2 months (April 2021), extended to infants <2 months (August 2023)
Administration		
Intrathecal; four loading doses over 2 months, then every 4 months	Intravenous; one time only	Oral; daily

AAV = adeno-associated virus; SMA = spinal muscular atrophy; SMN = survival motor neuron.

The aim of this study was to provide an overview of the evolving treatment landscape documented within SMArtCARE, which is a disease-specific registry collecting longitudinal real-world data in clinical routine since 2017. Data are collected across 84 participating centres in Germany, Austria and Switzerland. SMArtCARE was selected by the German Joint Federal Committee for routine data collection to assess the additional clinical benefit of OA and risdiplam.^16,17^ Participation in SMArtCARE is therefore mandatory for all centres prescribing these DMTs, ensuring near-complete and representative data capture across the treated patient population and providing a unique opportunity to analyse treatment decisions and their evolution over time.

## Materials and methods

Within the SMArtCARE registry, data are collected during routine patient visits and documented using standardized case report forms. Standardized physiotherapeutic assessments are performed to monitor the motor function of patients. Central ethics approval was obtained from the Ethics Committee of the University of Freiburg (EK-Freiburg 56/18), and the study is registered in the German Clinical Trial Register (DRKS00012699). For the present analysis, data extraction from the registry was performed on 24 February 2025.

All patients in the registry were included in the analysis. The main focus of the analysis was the DMT used as first-line treatment and whether there had been any changes of DMT during the course of treatment.

For the descriptive analysis, patients were divided into subgroups according to their treatment regimens. According to the classifications provided by Proud *et al*.,^18^ patients who changed DMT were classified as switchers if the duration of the first DMT was >3 months; patients were considered treatment bridgers if the duration of the first DMT was <3 months. Patients who started another DMT after treatment with OA were included in the subgroup ‘OA add-on’. Given that OA is a one-time infusion with potentially lifelong *SMN* gene expression, it is not possible to switch away from OA.^18^All patients in this group started with OA as the first DMT and subsequently had add-on therapy with nusinersen or risdiplam. Patients with an unknown DMT history that could not be verified by a data manager, and patients with concomitant use of two DMTs, were excluded from the descriptive analysis.

For a detailed subgroup analysis, we focused on patients administered with a monotherapy and thus excluded patients without a DMT and those in the OA add-on group. We compared treatment groups according to factors such as age at start of treatment, *SMN2* copy number, motor function (non-sitter, sitter or walker), the need for ventilator support or tube feeding, and the presence of scoliosis. Subgroups with ≤10 patients were excluded from the analysis, owing to the small sample sizes. Here, bridgers were assigned to the cohort corresponding to their second DMT, effectively grouping them with patients who had received that DMT initially and retained their first-line treatment. Transition plots were created to demonstrate changes in the treatment landscape over time across different subgroups. Patients receiving more than two types of treatment or no DMT and those with an unknown DMT history were excluded from the plots. Additional treatments in the OA add-on subgroup and bridging treatments were not considered. Patients were classified as NBS patients if the diagnosis was made presymptomatically by NBS or a positive family history.

## Results

Of the 2148 patients registered in the SMArtCARE registry, 2140 patients were included in the analysis. Eight patients were excluded owing to the concomitant use of risdiplam and nusinersen or an unknown history regarding the use of DMT.

A total of 1294 patients (60.5%) started with nusinersen as their first DMT, 514 patients (24.0%) with risdiplam and 243 patients (11.4%) with OA. Additionally, 89 patients (4.2%) were not treated with any DMT. The majority of patients (63.8%) remained on their initial DMT. Switches occurred primarily (45.2%) in the year of approval of the respective second DMT.

Among patients who started treatment with nusinersen, 46.6% remained on this therapy, whereas 38.9% changed to risdiplam and 14.5% transitioned to OA after the respective therapies became available. A few patients (2.4%) switched back to nusinersen after a switch from nusinersen to risdiplam. In the OA group, only 6.6% received a second DMT as add-on therapy. Risdiplam, being the most recently approved therapy, was continued by 82.5% of patients; 15.8% transitioned to OA, and 1.8% switched to nusinersen.

**Figure 1:**
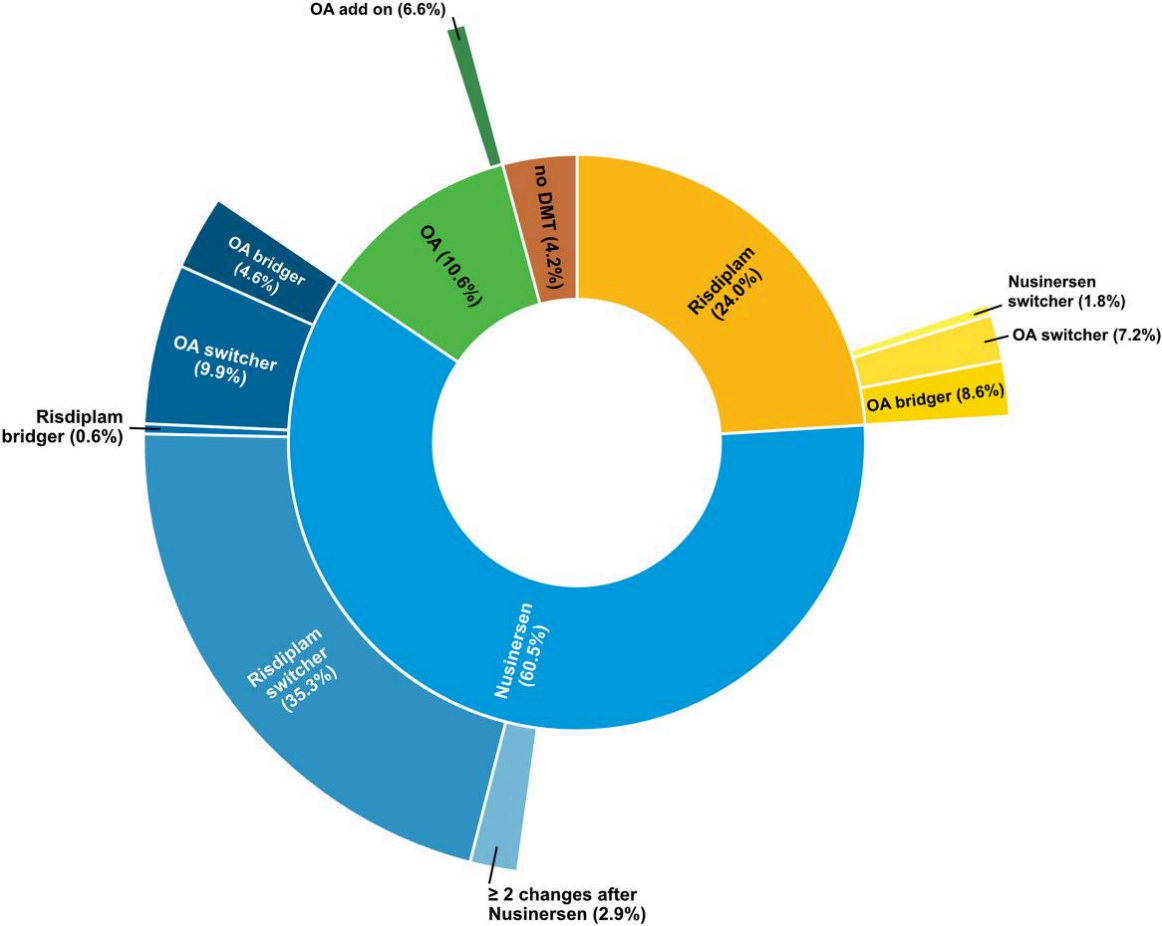
**Sunburst diagram showing treatment subgroups based on DMT sequence.** The *inner circle* represents the first DMT, and the *outer circle* represents the second DMT and, where applicable, the subgroup of patients receiving more than two different DMTs. Patients who changed DMT within 3 months of treatment initiation are categorized as bridgers; those who changed after >3 months are categorized as switchers. DMT = disease-modifying treatment; OA = onasemnogene abeparvovec.

An overview of initial treatment choices and subsequent therapy sequences is shown in Fig. 1.

### Patients retaining first-line treatment

Sex distribution was balanced in the nusinersen, risdiplam and OA subgroups, and the majority of patients had a homozygous deletion in *SMN1*. In accordance with the restricted approval of OA (Table 1), all patients in the OA cohort had up to three *SMN2* copies: 170 patients (51.4%) with up to two *SMN2* copies and 161 patients (48.6%) with three *SMN2* copies. In the nusinersen and risdiplam cohorts, the majority of patients had three *SMN2* copies [nusinersen, 236 patients (39.1%); risdiplam, 214 patients (49.5%)]. Patients with up to two *SMN2* copies or four *SMN2* copies showed a similar distribution in the nusinersen and risdiplam cohorts (Supplementary Table 1).

**Figure 2:**
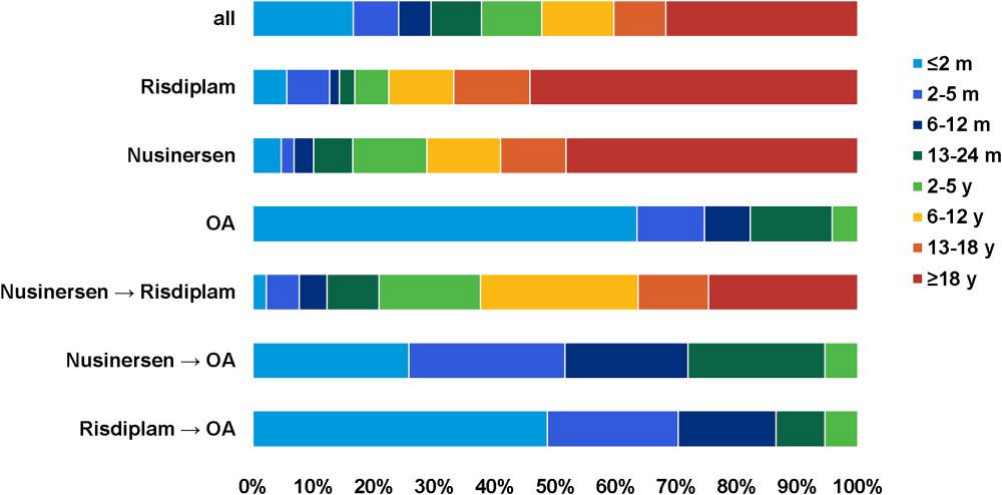
**Age distribution at initiation of first DMT across subgroups.** Patients categorized as bridgers were assigned to the cohort corresponding to their second DMT, grouping them with patients who received that therapy initially and remained on it. DMT = disease-modifying treatment; m = months; OA = onasemnogene abeparvovec; y = years.

Regarding the age at initiation of the first DMT, the majority of patients in the OA cohort started treatment at ≤2 months of age [210 patients (63.4%)], whereas these very young patients represented only a small percentage of the nusinersen [28 patients (4.6%)] and risdiplam [24 patients (5.6%)] cohorts. This difference was also seen in patients >18 years at initiation of the first DMT: 290 adult patients (48.1%) in the nusinersen subgroup and 232 adult patients (53.7%) in the risdiplam subgroup. As expected, no patients aged 6 years or older were included in the OA cohort. The distribution of age at the start of the first DMT is displayed in Fig. 2.

Symptom onset also varied: 95.9% of patients receiving nusinersen and 84.0% receiving risdiplam were symptomatic at treatment start, compared with 53.8% in the OA group.

Mainly owing to the earlier treatment initiation, 68.3% of patients in the OA cohort had not achieved any World Health Organization motor milestone prior to treatment, compared with 25.7% in the nusinersen group and 44.2% in the risdiplam group. A notable proportion in the latter two groups were ambulatory at treatment initiation: 35.0% for nusinersen and 19.4% for risdiplam.

At treatment initiation, the overall rate of patients requiring respiratory support or tube feeding was low, but higher in the nusinersen or risdiplam groups than in the OA group, reflecting a more advanced disease stage. Scoliosis was also more common in the nusinersen and risdiplam cohorts, probably owing to the older age at treatment initiation. Only a small number of patients in both groups had undergone scoliosis surgery before starting treatment.

Full demographic and clinical data are shown in Supplementary Table 1.

### Patients switching DMTs

Sex distribution was evenly balanced across the different switcher subgroups and similar to the cohorts with first-line DMT continuation. Most patients in the switcher subgroups had a homozygous deletion in *SMN1*. Regarding *SMN2* copy numbers, all but two patients who switched from either nusinersen or risdiplam to OA had up to three *SMN2* copies. Most patients (78.9%) who switched DMTs showed no change in motor milestones (neither gain nor loss) between the start of the first and the start of the second treatment (Table 2).

**Table 2:** Age and further characteristics of patients at switch to second disease-modifying treatment

Parameter	Nusinersen → risdiplam *n* = 457	Nusinersen → OA *n* = 128	Risdiplam → OA *n* = 37
Age at switch to second DMT			
≤2 months	0 (0.0%)	0 (0.0%)	0 (0.0%)
2–5 months	0 (0.0%)	0 (0.0%)	11 (29.7%)
6–12 months	0 (0.0%)	20 (15.6%)	12 (32.4%)
13–24 months	4 (0.9%)	46 (35.9%)	9 (24.3%)
2–5 years	50 (10.9%)	57 (44.5%)	5 (13.5%)
6–12 years	175 (38.3%)	5 (3.9%)	0 (0.0%)
13–18 years	89 (19.5%)	0 (0.0%)	0 (0.0%)
≥18 years	135 (29.5%)	0 (0.0%)	0 (0.0%)
Change of motor milestones between first and second DMT			
No change	394 (86.2%)	69 (53.9%)	28 (75.7%)
Sitting gained	37 (8.1%)	46 (35.9%)	9 (24.3%)
Sitting lost	4 (0.9%)	0 (0.0%)	0 (0.0%)
Walking gained	14 (3.1%)	13 (10.2%)	0 (0.0%)
Walking lost	4 (0.9%)	0 (0.0%)	0 (0.0%)
New ventilator support between first and second DMT	60 (13.1%)	30 (23.4%)	5 (13.5%)
New tube feeding between first and second DMT	33 (7.2%)	21 (16.4%)	6 (16.2%)
New scoliosis between first and second DMT	158 (34.6%)	45 (35.2%)	7 (18.9%)
Scoliosis surgery between first and second DMT	106 (32.2%)	1 (0.8%)	0 (0.0%)

DMT = disease-modifying treatment; OA = onasemnogene abeparvovec.

The largest group of switchers transitioned from nusinersen to risdiplam, with nearly all these patients being older than 2 years (≥18 years, 24.7%; 2–18 years, 73.1%; ≤2 years, 2.2%). In this group, 42.5% had undergone scoliosis surgery prior to the switch. The second largest group switched from nusinersen to OA, followed by patients who switched from risdiplam to OA. As expected, switches to OA occurred only in infants and young children. Given that nusinersen, OA and risdiplam were approved sequentially, the proportion of patients switching from risdiplam to OA has remained relatively low. The age distribution and clinical characteristics of the different switcher groups are shown in Fig. 2 and Table 2, respectively.

### Evolving treatment landscape over time

**Figure 3:**
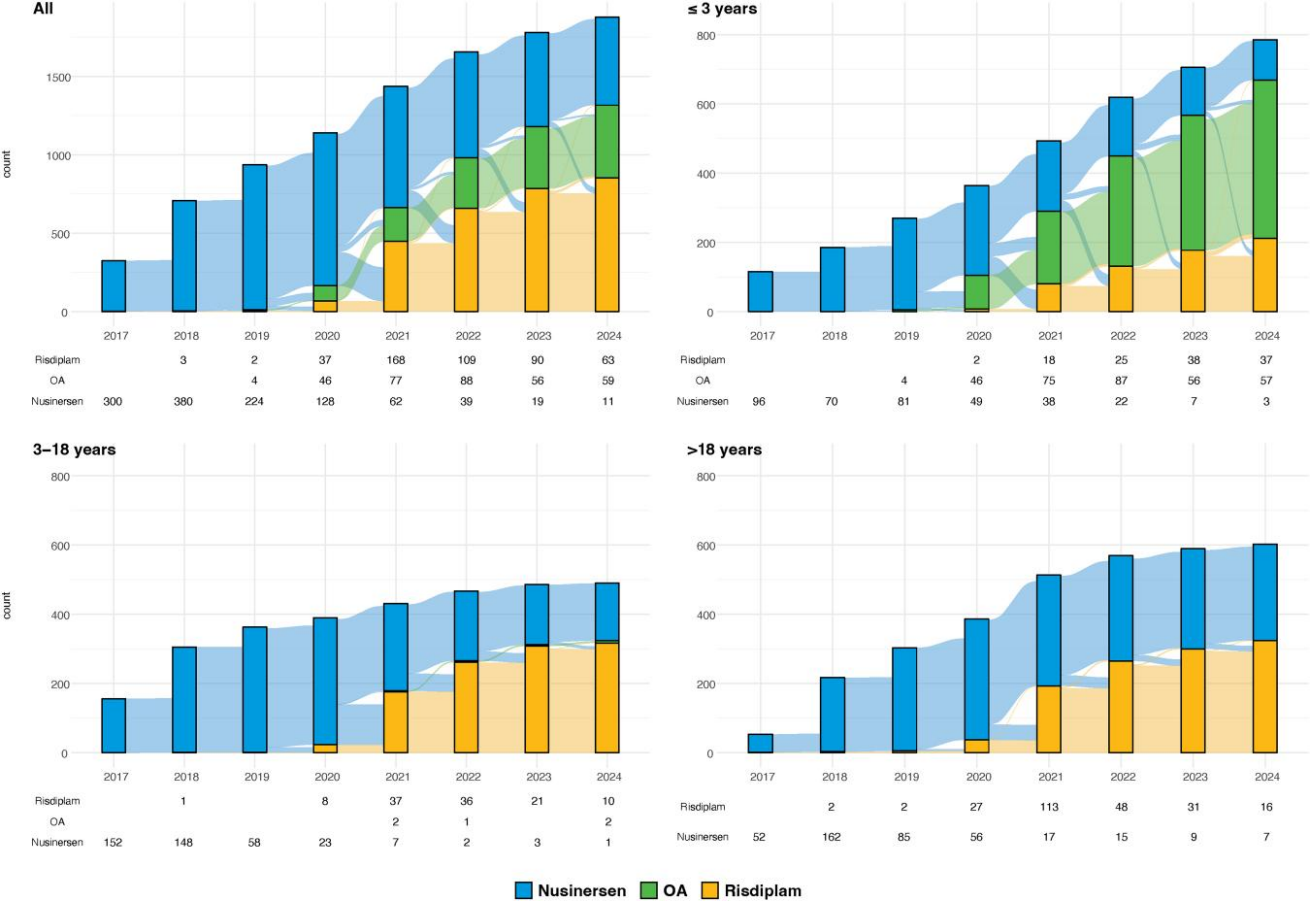
**Transition plots of DMTs and DMT switches, distributed by age at start of first DMT.** DMT = disease-modifying treatment; OA = onasemnogene abeparvovec.

Figure 3 illustrates the evolving treatment landscape between 2017 and 2024, stratified by age at initiation of the first DMT. Prior to 2020, nusinersen was the only available DMT. Most treatment switches occurred in the year of approval of a new DMT or shortly thereafter.

Among patients younger than 3 years, the most notable treatment shift was from nusinersen to OA, followed by patients shifting from nusinersen to risdiplam. In older children, a substantial number of patients switched from nusinersen to risdiplam, a trend that was also observed, albeit to a lesser extent, in adult patients. Following the approval of risdiplam, a notable number of previously untreated adults initiated therapy with risdiplam as their first DMT.

Although the majority of patients who initiated treatment with nusinersen remained on this drug, OA has more recently become the most common first-line treatment for children <3 years of age, while risdiplam has been used increasingly in older children and adults. By 2024, the adult patient population was equally divided between treatment with nusinersen and risdiplam.

**Figure 4:**
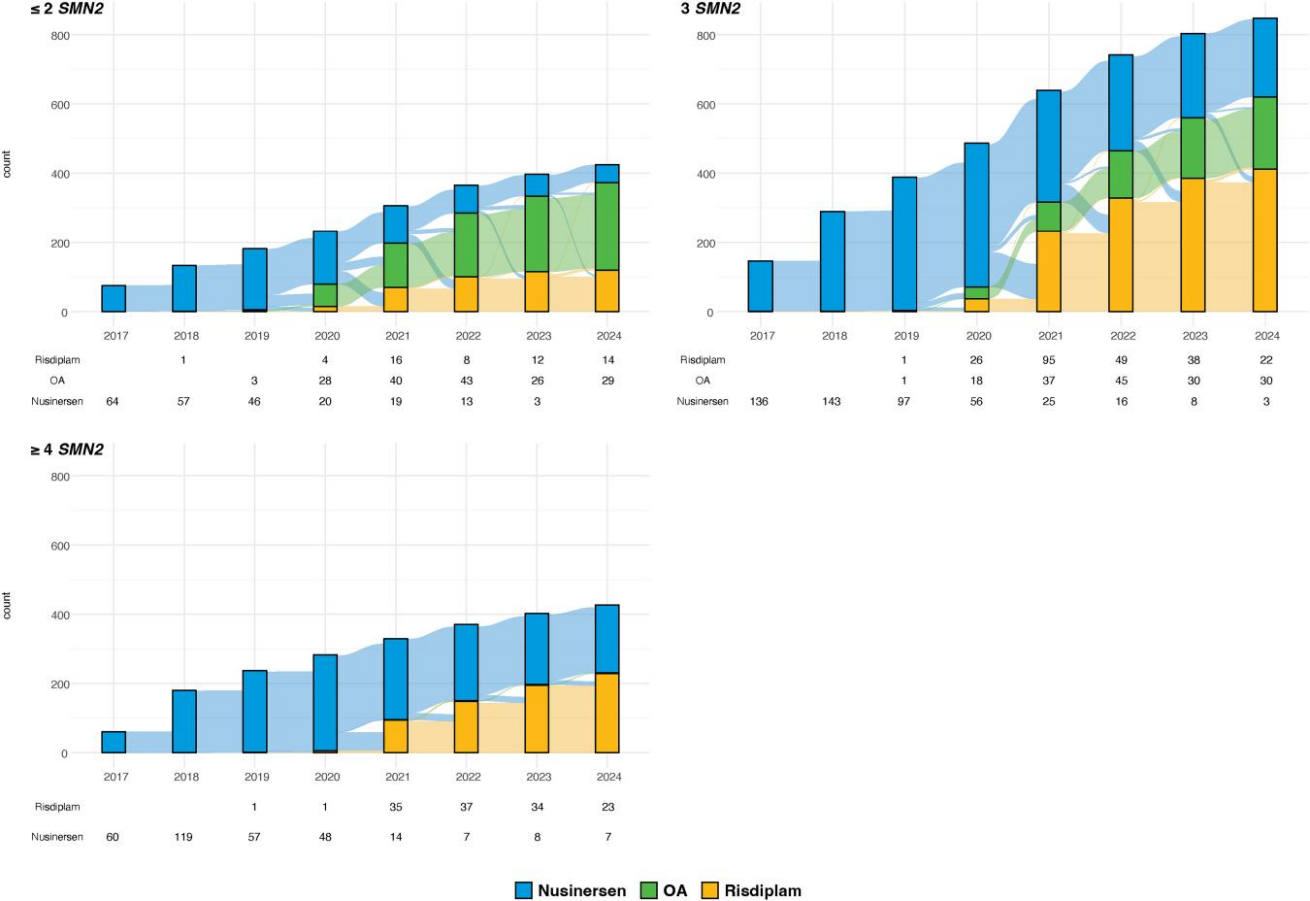
**Transition plots of DMTs and DMT switches, distributed by *SMN2* copy numbers.** DMT = disease-modifying treatment; OA = onasemnogene abeparvovec.

Stratifying patients and treatment transitions by *SMN2* copy number provides additional insights into treatment patterns (Fig. 4). Owing to its restricted approval, OA is only used as primary or secondary treatment in patients with up to three *SMN2* copies. In contrast, patients with at least four *SMN2* copies either remained on nusinersen or switched to risdiplam following its approval in 2021. In recent years, risdiplam has become the more commonly used first-line treatment in this group.

**Figure 5:**
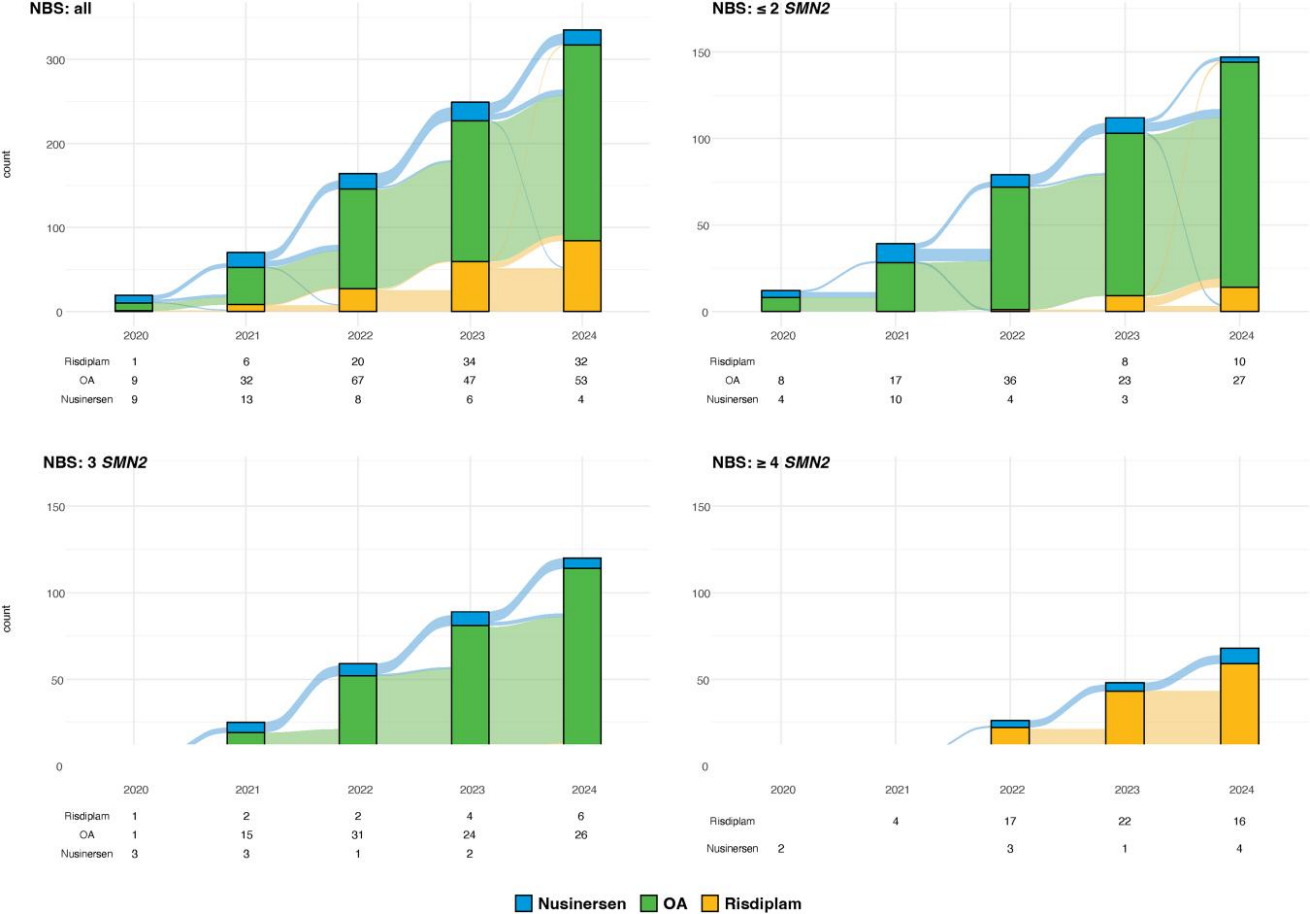
**Transition plots of DMTs and DMT switches in patients identified by NBS, distributed by *SMN2* copy numbers.** DMT = disease-modifying treatment; NBS = newborn screening programmes; OA = onasemnogene abeparvovec.

Figure 5 displays the use of DMTs among patients identified through NBS from 2020 to 2024, stratified by *SMN2* copy number. The number of new patients increased markedly over time owing to the rollout of NBS in participating countries. Patients with up to three *SMN2* copies exhibited similar treatment patterns: in 2020, they were treated with either OA or nusinersen. Over time, the use of nusinersen declined in these groups. By 2024, most patients were treated with OA, and among newly diagnosed patients OA was clearly preferred, followed by risdiplam in a small number of cases. In contrast, among patients with at least four *SMN2* copies, risdiplam has become the treatment of choice for most patients.

## Discussion

This study provides a comprehensive overview of real-world treatment decisions in a large and representative cohort of patients with SMA across all age groups, based on prospective data collection through the SMArtCARE registry. With >2100 registered patients, SMArtCARE is one of the largest SMA registries worldwide, and it offers broad coverage across almost all treatment centres in Germany, Austria and Switzerland. The registry began data collection in 2017, with the approval of the first DMT, and uniquely reflects treatment dynamics in a setting where all three EMA-approved therapies rapidly became available, typically without additional prescribing restrictions. This allows a relatively unbiased view of clinical practice and evolving treatment decision-making in response to regulatory approvals.

Analysing treatment patterns over time in SMA is inherently complex, because multiple interrelated factors influence both the disease course and therapeutic decisions. These include the sequential availability of DMTs, the age- and severity-dependent presentation of SMA, and the overlap between treatment effects and normal motor development in early childhood. Moreover, the introduction and expansion of NBS have led to a growing number of patients initiating treatment presymptomatically, further altering the clinical landscape. Despite these complexities, our analysis, using stratification by age and *SMN2* copy number, along with longitudinal transition analysis, reveals clear shifts in treatment preferences since the approval of the first DMT in 2017.

In infants and children <3 years of age, OA has become the treatment of choice. Its use is limited by EMA approval to patients with up to three *SMN2* copies and is further constrained by the increased risk of side effects in older and heavier patients. With the widespread implementation of NBS, most patients are now diagnosed within the first weeks of life, enabling very early treatment initiation, which might contribute to a more favourable safety profile. Although close monitoring with regular blood sampling is required, OA offers the advantage of a one-time gene addition therapy administered early in life. Given that any delay in treatment initiation should be avoided, particularly in symptomatic infants or those with low *SMN2* copy numbers, immediate initiation of risdiplam or nusinersen has recently been proposed as a bridging strategy until OA can be administered. This approach was also documented in a small number of patients (2.1%) within the SMArtCARE cohort who received risdiplam as bridging treatment. With one exception with an overlapping treatment of risdiplam for 4 weeks, all these children stopped treatment with risdiplam before receiving OA. Another strategy under discussion and currently explored in clinical trials is the use of risdiplam or nusinersen as an add-on therapy following administration of OA^19,20^; however, this has so far been observed rarely in our registry.

In older children (age at first treatment >3 years) and adults, many patients initially began treatment with nusinersen and continued it even after the approval of risdiplam, suggesting a high level of treatment satisfaction or perceived effectiveness.^21^ However, a considerable number of patients (30.0%) in this age group switched from nusinersen to risdiplam. Our analysis revealed that a significant proportion of these patients had scoliosis or had undergone spinal fusion prior to the switch. The prevalence of scoliosis was also higher in the risdiplam cohort compared with the nusinersen cohort. Given that intrathecal administration can be more challenging in the presence of scoliosis or spinal fusion, these factors are likely to have contributed to the selection of risdiplam in these cases. In a small observational study among 14 patients switching from nusinersen to risdiplam, all patients indicated the method of administration as a factor influencing their decision to switch DMT.^22^ Belančić *et al*.^23^ observed in their retrospective data analysis among 17 patients switching from nusinersen to risdiplam that difficulty in performing lumbar puncture was the main reason to switch in almost half the patients. In a brief report on nine patients switching from nusinersen to risdiplam, almost half the patients had spine surgery.^24^ Moreover O’Reilly *et al*.^25^ discovered in their retrospective data analysis that the main reasons for switching from nusinersen to risdiplam were related to spine complexity and intrathecal administration challenges.

In addition, our data indicate, given that SMA is rarely diagnosed in adulthood, that some adult patients who had previously deferred treatment might have opted to initiate therapy once risdiplam became available as an oral option. Currently, treatment among adults in the SMArtCARE cohort is almost evenly split between nusinersen and risdiplam. However, severely affected patients are more likely to choose risdiplam and, in comparison, more ambulatory patients receive treatment with nusinersen (Supplementary Table 1).

Although three DMTs have been approved and had been available for 4 years at the time of data extraction, still almost two-thirds of patients continued with their first DMT, underlining satisfaction with the effectiveness and application of DMT. Our analysis showed that most of the DMT switches occurred shortly after the approval of a new DMT, rather than being evenly distributed over time. This pattern suggests that switches might have been driven more by practical considerations, such as mode of administration or patient preference, or by theoretical expectations regarding the newly available therapy, rather than by a perceived decline in effectiveness of the prior treatment. Supporting this interpretation, many patients who switched DMTs showed stability in motor milestones, with gains also observed among some (Table 2).

Until now, there have been few publications describing the evolving treatment landscape over time in larger cohorts of patients with SMA. Proud *et al*.^18^ were the first to make a proposal on definitions for combination of DMTs in 2023 based on paths of treatment of 443 patients collected in the international RESTORE registry. In the study by Proud *et al*.,^18^ the proportion of patients using OA therapy was much higher (43.8%); consequently, the proportions of patients using nusinersen or risdiplam were significantly smaller (11.1% and 2.0%, respectively). Although RESTORE is a disease-specific registry, it was set up by Novartis with the primary aim of collecting post-approval data for treatment with OA—most likely causing a selection bias towards this treatment. An Australian registry-based study including 195 patients, published in 2024, found 88.9% of patients using nusinersen, 30% using risdiplam and 17.5% who had received OA at any time.^26^ In addition to registry-specific selection biases, country-specific restrictions to drug access can also significantly influence the observed treatment landscape. For instance, in the UK, a switch of DMT is possible only under specific circumstances, and the decision needs to be ratified by a national multidisciplinary panel.^25^ In Brazil, in contrast, DMT is funded only for patients with up to three *SMN2* copies or infants with SMA type 1.^27^ Moreover, the availability or absence of an NBS, hence whether mainly presymptomatic newborns or symptomatic infants and children are newly treated, influences treatment landscapes. The advantage of the cohort from German-speaking countries presented in this study is the comprehensive coverage of treated patients, enabled by mandatory data collection and the prompt availability of all three DMTs in accordance with their regulatory labels and without additional prescribing restrictions.

One limitation of our study is that we did not capture the specific reasons for switching DMTs. Although our data provide valuable insights into patient characteristics associated with treatment changes, further studies are needed to evaluate the motivations of physicians, patients and families when making decisions to switch therapy. Furthermore, this study does not include specific outcome measures to assess DMT effectiveness, nor does it permit direct comparisons between treatment regimens. Such analysis requires prespecified statistical methods to account for potential confounders and cannot be conducted meaningfully within the large and heterogeneous cohort presented in this landscape overview. Instead, they must be performed in well-defined subgroups based on clinical and genetic characteristics.

Another limitation is the likely underrepresentation of untreated patients in our cohort. It remains unclear why many individuals, particularly within the adult population, are living with SMA but are not in contact with specialized neuromuscular centres. These individuals might not be captured by registry-based data, owing to either lack of access or personal choice regarding treatment.

## Conclusion

In conclusion, the analysis of our large and representative SMArtCARE cohort reveals evolving treatment preferences that have developed over time, closely following the sequential approval of the three available DMTs. Although patients who initiated treatment when only nusinersen was available often continue this therapy, newer treatments, such as risdiplam and OA, have increasingly become the preferred options, particularly among newly diagnosed patients identified through NBS. In the absence of head-to-head efficacy data, our findings suggest that treatment decisions in clinical practice are largely influenced by practical considerations, including the mode of administration, regulatory restrictions, and patient or caregiver preferences. The availability of a one-time gene therapy, the burden of intrathecal administration, and the convenience of oral treatment are likely to be key drivers in this dynamic landscape. As more data become available, particularly on long-term outcomes, these evolving patterns will continue to shape individualized treatment strategies for patients with SMA.

## Supplementary Material

awaf472_Supplementary_Data

## Data Availability

All data included in this analysis are recorded in the SMArtCARE registry. Data can be obtained (anonymized and aggregated) upon request and approval by the SMArtCARE steering committee.

## Appendix 1

### SMArtCARE study group

Pascal Martin, Christoph Neuwirth, Andreas Merkenschlager, René Günther, Mathias Müller, Regina Trollmann, Gert Wiegand, Sabine Illsinger, Omar Keritam, Matthias Baumann, Sarah Bernsen, Claudia Weiß, Gerd Meyer zu Hörste, Matthias Türk, Daniel Zeller, Thomas M. K. Völkl, Uta Diebold, Verena Haug, Wolfgang Löscher, Dorothea Holzwarth, Mareike Schimmel, Burkhard Stüve, Karin Wabnegger, Ralf A. Husain, Oliver Schwartz, Matthias Vorgerd, Eva Matzker, Gilbert Wunderlich, Raffi Topakian, Eckard Hamelmann, Anette Schwerin-Nagel, Marina Flotats-Bastardas, Astrid Blaschek, Christof Reihle, Astrid Bertsche, Ruth Janßen, Andreas Merkenschlager, Sandra Nebgen, Martin Smitka, Andreas Ziegler, Manuela Theophil, Ruth Janßen, Moritz Metelmann, Birgit Kauffmann, Andreas Hahn, Gudrun Schreiber, Anette Schwerin-Nagel, Astrid Eisenkölbl, Martin Winterholler, Eva Stögmann, Veronka Horber, Friedrich Ebinger, Tobias Geis, Martin Fleger, Jessika Johannsen, Meike Bettina Göricke, Veronika Pilshofer, Maren Fuchs, Paul Lingor, Alexander Mensch, Robert Steinbach, Sebastian Friedrich, Julian Großkreutz, Zeljko Uzelac, Friederike Sophie Kohlmann, Harald Faninger, Simone Mahal, Christoph Korenke, Harald Binder, Katharina Dörnbrack, Jasmin Bischofberger, Janina Gburek-Augustat, Hanna Sophie Lapp, Tanja Zindler, Bettina Behring, Miriam Hiebeler, Hans Hartmann, Benjamin Stolte, Bernhard Fasching, Christian Lechner, Patrick Weydt, Joanna Schneider, Sarah Wiethoff, Julia Bellut, Johannes C. Stoffels, Antje Schmidt, Corrine G. C. Horlings, Alexandra Klotz, Daniela Angelova-Toshkina, Sabine Knafl-Slamanig, Daniela Steuernagel, Hélène Guillemot, Melanie Drabant, Kathrin Hornung, Hormos Salimi Dafsari, Petra Müller, Georg Classen, Michael Grässl, Viola Horneff, Sarah Braun, Josefine Lendeckel, Oliver Summ, Janina Gburek-Augustat, Ilona Krauspe-Stübecke, Maja von der Hagen, Michal Fischer, Klaus Goldhahn, Oliver Summ, Christa-Caroline Bergner, Adela Della Marina, Marieke Ziegler, Omar Keritam, Kyriakos Martakis, Bernd Wilken, Michael Grässl, Manuel Pühringer, Christophe Rauch, Hanna Küpper, Julia Maren Lüttgenau, Marion Pilz, Jonas Denecke, Melanie Haage-Brüning, Kathrin Mörtlbauer, Franziska Grünberg-Lemi, Marcus Deschauer, Caroline Deborah Stapf, Annekathrin Rödiger, Cornelia Voigt-Müller, Janina von der Gablentz, Beate Muschner, Ronny Nebelung, Alina Sapranidis, Magdalena Gosk-Tomek, Heike Losch, Maren Hackenberg, Alexandra Giese, Ursula Schneider Rosinger, Sybille Döhler, Maria Rehfeldt, Stephanie Schüssler, Stephan Wenninger, Barbara Ramadan, Svenja Neuhoff, Gudrun Zulehner, Rachel Fabian, Angela Kaindl, Johannes Flammer, Brigitte Brauner, Carmen Hollerauer, Tajana Brunckhorst, Anna Hotter, Gabriel Dworschak, Lisa Jung, Sabine Borowski, Timo Deba, Anne-Katrin Güttsches, Lisa Starke, Jürgen-Christoph von Kleist-Retzow, Ina Krahwinkler, Burkhard Gess, Lukas Neubauer, Michael Zemlin, Wolfgang Müller-Felber, Markus Blankenburg, Josefin Grabow, Philipp Hoth, Ilka Lehnert, Georg Friedrich Hoffmann, Arpad von Moers, Philipp Hoth, Petra Baum, Nina Rademacher, Ulf Hustedt, Bernhard Fasching, Jolanta Heinzinger, Magnus Ruf, Barbara Plecko, Marie-Luise Drax, Frank Kerling, Nadja Kaiser, Eleni Chatzizisi, Joenna Driemeyer, Meret Tabea Dreyer, Elke Pernegger, Isabell Cordts, Ilka Schneider, Benjamin Ilse, Sara-Maria Simon, Bernd Friedrich, Kurt Wollinsky, Janina Gburek-Augustat, Franz-Philipp Brändle, Anna Wiesenhofer, Iris Marquardt, Clemens Schächter, Christine Mauz, Christina Knellwolf, Maren Freigang, Raphael Seebröker, Simone Thiele, Melina Schlag, Jakob Rath, Margrit Koglin, Lisa Valerie Bitzan, Christine Leypold, Florian Junge, Katrin Schüler, Franziska Busch, Sabrina Geissler, Heike de Vries, Katja Köbbing, Ute Weyen, Ioanna Angeli, Barbara Plecko, Kerstin Böcking, Moritz Tacke, Michael Schroth, Frauke Ehrhardt, Monika Meyer, Nicole Claus, Stefan Kölker, Kathrin Bühner, Monika Meyer, Jennifer Rolack, Britta Holtkamp, Inka Brandes, Jakob Rath, Rahel Schuler, Elisabeth Steiner, Martin Drechsel, Dorothee Fütterer, Patricia Müller, Paula Steffens, Ulrike Wolf, Tanja Neumair, Marianne Rösner, Anna Katharina Koelsch, Almut Fritsch, Eva Jung, Clemens Runge, Kornelia Kreiser, Andreas Merkenschlager, Susanne Grinzinger, Yvonne Lechner, Renate Peters, Max Behrens, Adrian Tassoni, Zylfije Dibrani, Marian Kollaske, Laura Stimmer, Jaquelina Lipka, Martin Krenn, Martina Delitz, Alexandra Wagner, Katja D’Amico-Hofmann, Anna Resch, Lisa Allenstein, Stephanie Molitor, Peter Huppke, Andor Horváth, Jakob Wefers, Julia Turowski, Sabine Hettrich, Iris Hannibal, Lutz Dondit, Aglaia Lütjens, Stefan Kappel, Sandy Förster, Sabine Specht, Katharin Müller-Kaempffer, Stefan Kappel, Barbara Andres, Krisitin Kook, Martin Krenn, Andrea Hackemer, Doris Roland-Schäfer, Ingo Conze, Deike Weiss, Mario Müller, Anna Elmecker, Petra Rau, Thomas Kendzierski, Uta Smesny, Nikolai Jung, Astri Fromm, Johannes Dorst, Henriette Kiep, Christian Rauscher, Katia Vettori, Tobias Linden, Tim Kampowski, Anja Boegner, Eva Malm, Fritz Zimprich, Barbara Schwegmann, Vladimir Dukic, Rike Remmert, Christa Bretschneider, Barbara Fiedler, Corinna Rademacher, Loreen Plugge, Sybille Stephan-Lutter, Lena Manssen, Eva Wendel, Martina Neininger, Anja Meenken, Moritz Niesert, Annika Meenken, Nina Schlaghecke, Meike Kahrs, Fritz Zimprich, Lena Ruß, Eva Jansen, Heidi Starke, Jörg Tiedemann, Verena Angermair, Vincent Gmeiner, Sebastian Plutz, Anna-Lena Fleischer, Sabine Wider, Kristin Loyal, Victoria Göbel, Alexander Bobe, Anna-Maria Gaese, Katharina Längle, Birgitt Moed, Franziska Wenzel, Katharina Wagner, Peter Reilich, Thomas Dörnen, Corinna Stoltenburg, Hanna Reinke-Hermer, Sindy Becker, Eva Johann to Settel, Karsten Krause, Christine Sprengart, Birgit Warken-Madelung, Daniela Banholzer, Katja Koch, Urania Kotzaeridou, Katja Koch, Jana Barluschke, Kirsten Fündeling-Tielitz, Gudrun Zulehner, Katharina Unverfehrt, Esther Maihöfer, Ricarda von Heynitz, Max Obenauf, Nadin Fedtke, Juliane Hug, Mohammad Kalaf, Julia Seifert, Ramona Menzel, Mirjam Spitzinger, Anna Hüpper, Shalin Nerkamp, Lukas Nalbach, Beate Schlotter-Weigel, Zoé Messerli, Anja Müller, Geerthe Margriet Balk, Irene Lange, Nicole Groß, Annika Roser-Unruh, Elke Preisendanz, Jessica Ribke, Annegret Hoevel, Jessica Ribke, Corinna Doege, Daniel Bormann, Heidrun Krämer-Best, Birgit Meßmer, Barbara Vogel, David Strube, Saskia Beyer, Matthias Eckenweiler, Maj-Britt Bartels, Aneliya Simeonova Eroglu, Elena Gantschew, Elke Tiefenthaler, Nora Maier, Janina Fürup, David Leibensperger, Laura Kahle, Sebahattin Cirak, Sibylle Vogt, Sabine Stein.
