# Supplementary material for: Treatment evolution in spinal muscular atrophy: insights from the SMArtCARE registry
Source: Brain. 2025 Dec 23;149(3):818–27. doi: 10.1093/brain/awaf472 (PMC13016938; doi:10.1093/brain/awaf472)
Supplement: awaf472_Supplementary_Data [file awaf472_supplementary_data.pdf]

Supplemental table 1: Clinical data of all patients at start of the 1st DMT

		all	risdiplam	nusinersen	OA	nusinersen → risdiplam	nusinersen → OA	risdiplam → OA
n		2,004	432	603	331	457	128	37
**Sex**	**male**	1,043 (52.0%)	213 (49.3%)	350 (58.0%)	151 (45.6%)	229 (50.1%)	72 (56.3%)	16 (43.2%)
SMN1	**deletion**	1,834 (91.5%)	386 (89.4%)	545 (90.4%)	325 (98.2%)	402 (88.0%)	125 (97.7%)	35 (94.6%)
	**point mutation**	50 (2.5%)	13 (3.0%)	16 (2.7%)	5 (1.5%)	12 (2.6%)	3 (2.3%)	1 (2.7%)
SMN2	**≤2**	472 (23.6%)	33 (7.6%)	62 (10.3%)	170 (51.4%)	89 (19.5%)	83 (64.8%)	20 (54.1%)
	**3**	884 (44.1%)	214 (49.5%)	236 (39.1%)	161 (48.6%)	213 (46.6%)	44 (34.4%)	16 (43.2%)
	**4**	397 (19.8%)	121 (28.0%)	189 (31.3%)	0 (0.0%)	85 (18.6%)	1 (0.8%)	1 (2.7%)
	**≥5**	57 (2.8%)	10 (2.3%)	23 (3.8%)	0 (0.0%)	23 (5.0%)	0 (0.0%)	0 (0.0%)
**Age at start 1st DMT**	**≤2 months**	331 (16.5%)	24 (5.6%)	28 (4.6%)	210 (63.4%)	10 (2.2%)	33 (25.8%)	18 (48.6%)
	**2-5 months**	150 (7.5%)	30 (6.9%)	13 (2.2%)	37 (11.2%)	25 (5.5%)	33 (25.8%)	8 (21.6%)
	**6-12 months**	107 (5.3%)	7 (1.6%)	19 (3.2%)	25 (7.6%)	21 (4.6%)	26 (20.3%)	6 (16.2%)
	**13-24 months**	167 (8.3%)	11 (2.5%)	39 (6.5%)	45 (13.6%)	39 (8.5%)	29 (22.7%)	3 (8.1%)
	**2-5 years**	198 (9.9%)	24 (5.6%)	74 (12.3%)	14 (4.2%)	77 (16.8%)	7 (5.5%)	2 (5.4%)
	**6-12 years**	238 (11.9%)	46 (10.6%)	73 (12.1%)	0 (0.0%)	119 (26.0%)	0 (0.0%)	0 (0.0%)
	**13-18 years**	172 (8.6%)	54 (12.5%)	65 (10.8%)	0 (0.0%)	53 (11.6%)	0 (0.0%)	0 (0.0%)
	**≥18 years**	635 (31.7%)	232 (53.7%)	290 (48.1%)	0 (0.0%)	113 (24.7%)	0 (0.0%)	0 (0.0%)
**clinically silent at start 1st DMT**	**clinically silent**	287 (35.4%)	65 (15.0%)	23 (3.8%)	153 (46.2%)	13 (2.8%)	17 (13.3%)	15 (40.5%)
	**symptomatic**	1,711 (85.4%)	363 (84.0%)	578 (95.9%)	178 (53.8%)	444 (97.2%)	111 (86.7%)	22 (59.5%)
**motor milestone at start 1st DMT**	**no**	897 (44.8%)	191 (44.2%)	155 (25.7%)	226 (68.3%)	177 (38.7%)	99 (77.3%)	33 (89.2%)
	**sitting**	600 (29.9%)	133 (30.8%)	228 (37.8%)	42 (12.7%)	171 (37.4%)	24 (18.8%)	2 (5.4%)
	**walking**	389 (19.4%)	84 (19.4%)	211 (35.0%)	9 (2.7%)	82 (17.9%)	1 (0.8%)	2 (5.4%)
**ventilator support at start 1st DMT**		356 (17.1%)	107 (24.8%)	93 (15.4%)	13 (3.9%)	114 (24.9%)	12 (9.4%)	3 (8.1%)
**tube feeding at start 1st DMT**		148 (7.4%)	33 (7.6%)	29 (4.8%)	13 (3.9%)	64 (14.0%)	9 (7.0%)	0 (0.0%)
**scoliosis at start 1st DMT**		610 (30.4%)	195 (45.1%)	213 (35.3%)	3 (0.9%)	194 (42.5%)	4 (3.1%)	0 (0.0%)
**scoliosis surgery before start 1st DMT**		42 (2.1%)	17 (3.9%)	13 (2.2%)	0 (0.0%)	12 (2.6%)	0 (0.0%)	0 (0.0%)

Abbreviations: DMT = disease modifying treatment; OA = onasemnogene abeparvovec
